# Growth hormone activates PI3K/Akt signaling and inhibits ROS accumulation and apoptosis in granulosa cells of patients with polycystic ovary syndrome

**DOI:** 10.1186/s12958-020-00677-x

**Published:** 2020-12-07

**Authors:** Yan Gong, Shan Luo, Ping Fan, Huili Zhu, Yujing Li, Wei Huang

**Affiliations:** 1 Department of Obstetrics and Gynecology, West China Second University Hospital of Sichuan University, Chengdu, Sichuan People’s Republic of China; 2 Key Laboratory of Birth Defects and Related Diseases of Women and Children, Ministry of Education, Chengdu, Sichuan People’s Republic of China; 3 Reproductive Medicine Center, Sichuan Provincial Women’s and Children’s Hospital, The Affiliated Women’s and children’s Hospital of Chengdu Medical College, Chengdu, Sichuan People’s Republic of China; 4 Laboratory of Genetic Disease and Perinatal Medicine, Key Laboratory of Birth Defects and Related Diseases of Women and Children, Ministry of Education, Chengdu, Sichuan People’s Republic of China; 5 Department of Reproductive Medicine, West China Second University Hospital of Sichuan University, #1416 Chenglong Road, JinJiang District, Chengdu, Sichuan 610041 People’s Republic of China

**Keywords:** Polycystic ovary syndrome, Growth hormone, Reactive oxygen species, Apoptosis, PI3K/Akt signaling

## Abstract

**Background:**

It is reported that growth hormone (GH) can alleviate oxidative stress (OS) induced apoptosis in some types of cells by activating the PI3K/Akt signaling pathway. This study investigated the role and underlying mechanism of GH in OS and apoptosis in granulosa cells (GCs) of patients with polycystic ovary syndrome (PCOS).

**Methods:**

Primary GCs were collected from patients with and without PCOS (controls, *n* = 32) during oocyte retrieval. The patients with PCOS were randomly assigned to take GH treatment (PCOS-GH, *n* = 30) or without GH treatment (PCOS-C, *n* = 31). Reactive oxygen species (ROS) level was determined by spectrophotometry and fluorescence microscopy. GC apoptosis and mitochondrial membrane potential (MMP) were detected by Annexin V-FITC/PI double-staining and JC-1 staining, respectively (flow cytometry). The expression of apoptosis-related genes and proteins involved in PI3K/Akt signaling was determined by quantitative reverse-transcription polymerase chain reaction and western blotting, while active caspase-9 and caspase-3 levels of GCs were determined by enzyme-linked immunosorbent assay.

**Results:**

Our study found that in GCs of the PCOS-GH group, the ROS levels and apoptotic rates were significantly decreased, whereas MMP was significantly increased when compared to those in the PCOS-C group (*P* < 0.05). The mRNA levels of *FOXO1*, *Bax*, *caspase-9*, and *caspase-3* were significantly decreased, whereas *Bcl-2* was increased in GCs of the PCOS-GH group than those in the PCOS-C group (*P* < 0.05). The protein levels of FOXO1, Bax, cleaved caspase-9/caspase-9 and cleaved caspase-3/caspase-3 were decreased, whereas p-PI3K/PI3K, p-Akt/Akt, p-FOXO1 and Bcl-2 were increased in GCs of the PCOS-GH group, compared with those in the PCOS-C group (*P* < 0.05).

**Conclusion:**

OS induced apoptosis and downregulated the PI3K/Akt signaling pathway in patients with PCOS. GH could alleviate apoptosis and activate the PI3K/Akt signaling pathway.

**Clinical trial registration number:**

Chinese Clinical Trial Registry. ChiCTR1800019437. Prospectively registered on October 20, 2018.

## Introduction

Polycystic ovarian syndrome (PCOS) is the most common endocrinopathy that affects 5–10% women of reproductive age. The characteristics of PCOS are hyperandrogenemia, polycystic ovaries, and/or ovulation dysfunction [1]. Chronic anovulation results in infertility; and in vitro fertilization (IVF) and embryo transfer (ET) is the common treatment when the patients failed pregnancy with ovulation induction.

Reactive oxygen species (ROS) and/or reactive nitrogen species (RNS) are produced in many physiological processes. The antioxidant mechanism existing in the body can maintain ROS and RNS at low concentrations, which is beneficial for normal cell function [2]. Oxidative stress (OS), resulting from an imbalance between radicals and antioxidant defense, has been found to be a main pathophysiological mechanism in various human diseases. The excessive ROS can induce mitochondrial mediated apoptosis [3]. It is reported that OS, mitochondrial dysfunction, and OS-induced apoptosis are present in the granulosa cells (GCs) of PCOS [4–6]. Through bidirectional communication, GCs play important role in oocyte maturation, fertilization, and subsequent implantation [7, 8]. Therefore, apoptosis in GCs is associated with poor oocyte quality and IVF outcomes in patients with PCOS [6, 8].

Phosphatidylinositol 3-kinase (PI3K) signaling is a fundamental pathway for the regulation of cell proliferation, survival, migration, and metabolism in a variety of physiological and pathological processes. Recent studies in human and mouse confirmed that the PI3K/Akt signaling plays a crucial role in the regulation of GC growth and apoptosis during follicular development [9–11]. Forkhead box O (*FOXO*) transcription factors are downstream targets of PI3K/Akt. Phosphorylation of FOXOs by p-Akt inhibits transcriptional functions of FOXOs and contributes to cell survival, growth and proliferation [12]. As a member of *FOXOs* family, *FOXO1* plays a pivotal role in up-regulating the expression of downstream pro-apoptosis genes, then induces GCs apoptosis via caspase family induced mitochondrial pathway [13–16].

Growth hormone (GH) can reduce OS-induced apoptosis in some types of cells including vascular endothelium, cardiomyocytes, and neural and skeletal muscle cells by activating the PI3K/Akt signaling pathway [17–20]. Hence, GH has been widely applied to treat pathologies associated with OS [20]. GH receptors are expressed in human GCs and oocytes. Exogenous GH administration alleviates mitochondrial dysfunction and improves oocyte quality and IVF outcomes among older women and/or patients with poor ovarian response [21].

PCOS is a disease involving multiple genes and environmental factors [22]. Microarray data of GCs from patients with PCOS indicated that the markedly changed genes are mainly related to diabetes, inflammation, and OS [23]. The PI3K/Akt signaling pathway is dysregulated in both patients with PCOS and animal models of PCOS [24, 25]. Activation of the PI3K/Akt signaling pathway can reduce apoptosis induced by downstream signaling molecules [9–11], and consequently, not only protect GCs from OS injury but also improve oocyte quality and IVF outcomes [9]. However, the antioxidant effects of GH in GCs of patients with PCOS and related signaling pathways have not been investigated yet. Therefore, this study investigated the effects of GH on ROS levels, apoptosis of GCs, and the PI3K/Akt signaling pathway.

## Materials and methods

### Clinical samples

From November 2018 to November 2019, patients with PCOS (aged 22–36 years) diagnosed according to the Rotterdam criteria [26] were recruited, then further according to computer-generated random numbers randomly assigned into two groups: one group took GH treatment during controlled ovarian stimulation (COS) as PCOS-GH group; the other group had no GH treatment as PCOS-C group. Written informed consent was obtained from each participant. The study also conforms to the Declaration of Helsinki for Medical Research involving Human Subjects (2013 revision). Age-matched women of tubal infertility (aged 25–37 years) who underwent in vitro fertilization and embryo transfer (IVF-ET) were recruited as non-PCOS controls. Patients with hydrosalpinx, systemic lupus erythematosus, or sicca syndrome; uncontrolled endocrinopathy such as diabetes, hyperthyroidism, hypothyroidism, and hyperprolactinemia; or currently taking anti-OS medicine such as vitamin E, vitamin C, and Coenzyme Q10 were excluded from the study.

Medical history such as menstrual cycle regularity, duration of infertility, and treatment was collected from all the participants. Physical examinations included measurements of height, body weight, waist circumference, and hip circumference. Body weight index (BMI) was calculated as weight divided by height squared (kg/m^2^). The waist-to-hip ratio (WHR) was calculated as the waist circumference divided by the hip circumference. Androgen-related symptoms of hirsutism and acne were evaluated as previously reported [27, 28]. Plasma glucose, estradiol (E_2_), progesterone (P), total testosterone (TT), luteinizing hormone (LH), follicle-stimulation hormone (FSH), sex hormone binding globulin (SHBG), and fasting insulin (FINS) levels were measured as reported previously. The free androgen index (FAI) was calculated as TT (nmol/L)/SHBG (nmol/L) × 100. The homeostasis model assessment (HOMA-IR) index was calculated as fasting glucose (mmol/L) × fasting insulin (mIU/L)/22.5. The intra- and inter-assay coefficients of variation for these values were < 5 and < 10%, respectively.

COS regimen was gonadotropin-releasing hormone antagonist protocol for all participants. Recombined follicular stimulation hormone (rFSH) (Gonal-F; Merck-Serono KGaA., Darmstadt, Germany) was administered starting from day 2 of the menstrual cycle. The dose of rFSH was adjusted according to follicular growth. In the PCOS-GH group, patients were subcutaneously administered with 4 IU/d of recombinant human GH (Jintropin, Changchun GeneScience Pharmaceutical Co., Ltd., Changchun, Jilin, China) until the trigger day. Cetrorelix (Cetrotide; Merck-Serono KGaA.) was administered when one of the below criteria was matched: serum E_2_ > 300 pg/mL, leading follicle diameter reached 13–14 mm, LH > 10 IU/L. Recombinant human chorionic gonadotropin (Ovitrelle®; Merck-Serono KGaA., Darmstadt, Germany) was administered as the trigger when the diameters of at least two follicles reached ≥18 mm. After 36 h, oocytes were retrieved under transvaginal ultrasound guidance. During oocyte retrieval, primary GCs in follicle fluid (FF) were collected.

### Primary GC isolation

During oocyte retrieval, FF was collected from follicles with a diameter ≥ 16 mm measured on the retrieval day, then immediately separated by centrifugation at 700×*g* for 5 min at room temperature. The precipitates were suspended in left 2 ml of FF and gently layered into 3 mL of 50% lymphocyte separation medium (Solarbio Science and Technology Corporation, Beijing, China). After centrifugation at 700×*g* for 10 min at room temperature to remove red blood cells and debris, GCs layered at the interface of the gradient were collected and washed twice with 5 mL of phosphate-buffered saline (Nanjing KeyGen Biotech. Co., Ltd**.,** Nanjing, Jiangsu, China). The residual red blood cells were further removed using red blood cell lysis buffer (Solarbio Science and Technology Corporation). GCs from each patient were collected separately and considered as one sample. One portion of GCs was immediately examined intracellular ROS levels, mitochondrial membrane potential (MMP), and apoptosis; the remaining GCs were stored at − 80 °C refrigerator.

### Detection of intracellular ROS levels

ROS generation in GCs was measured using 2′,7′-diclorodihydrofluorescein di-acetate (H2-DCFDA) method by ROS assay kit (Beyotime Biotechnology Co., Ltd., Shanghai, China). Briefly, GCs were resuspended in PBS and incubated with 10 μM H2-DCFDA in the dark for 25 min at 37 °C, and then incubated with 10 μg/mL 4′,6-diamidino-2-phenylindole (DAPI) (NeoFroxx, Frankfurt, Germany) for 5 min. After washed three times with PBS, GCs suspensions were added to glass slides, and examined by fluorescence microscopy (Olympus Corporation, Tokyo, Japan). The examination wavelength was 488 nm, and the emission wavelength was 525 nm.

Similar with the above protocol but without DAPI, NanoDrop UV-Vis spectrophotometry (Thermo Scientific, MA, USA) was used to measure the intracellular ROS level of GCs. The relative ROS levels were presented as the fluorescence intensity of the PCOS group relative to that of non-PCOS controls.

### Apoptosis assay

Apoptosis of GCs were detected using the Annexin V-FITC apoptosis detection kits (KeyGEN Bio TECH Co., Ltd.). Briefly, 1 × 10 ^5^/ Test of GCs were resuspended in 500 μL binding buffer, then labeled with Annexin V-FITC (5 μL) and propidium iodide (PI) (5 μL) for 15 min in the dark at room temperature. After 1 h, the green (Annexin V-FITC) and red (PI) fluorescence were examined by flow cytometry (MilliporeSigma Co., Ltd., Burlington, MA, USA). The examination wavelength was 488 nm, and the emission wavelength was 530 nm.

### Detection of MMP

The MMP of GCs was examined using JC-1 Apoptosis Detection Kits (KeyGEN Bio TECH Co., Ltd.). In brief, 1 × 10 ^5^/ Test of GCs were resuspended and incubated with 500 μL JC-1 reagent solution at 37 °C in the dark for 15 min. JC-1 accumulates in functional mitochondria with high mitochondrial membrane potential (ΔΨm) and forms aggregates that emit red fluorescence. When mitochondrial transmembrane potential is depolarized with low ΔΨm, JC-1 releases from the mitochondria and forms monomers that emit green fluorescence. After washed two times with incubation buffer, the green and red fluorescence were examined by flow cytometry (MilliporeSigma Co., Ltd., Burlington, MA, USA). The examination wavelength was 488 nm, and the emission wavelength was 530 nm.

### Reverse-transcription and quantitative real-time polymerase chain reactions (RT-qPCR)

Frozen GCs were rapidly thawed and total RNA was isolated using the RNAprep Pure Micro Kit (Tiangen Biotech Co., Ltd., Beijing, China). The quality of RNA was checked at an absorbance of 260 nm/280 nm by Nanodrop-2000 (ThermoFisher Scientific, Waltham, MA, USA). Total RNA was reverse transcribed to cDNA using the PrimeScript™ RT reagent kit with gDNA Eraser (TaKaRa, Tokyo, Japan). Polymerase chain reaction (PCR) was performed using TB Green™ Premix Ex Taq™ II (TaKaRa) on a CFX96 real-time PCR detection system (Bio-Rad, Hercules, CA, USA) as follows: 95 °C for 30 s, followed by 40 cycles of 95 °C for 10 s, 60 °C for 30 s, and 65 °C for 5 s. The PCR system (20 μL) comprised RNase free dH_2_O (6.4 μL), cDNA (2 μL), forward primer (0.8 μL), reverse primer (0.8 μL) and 2 × TB Green Premix Ex Taq II (10 μL). All PCR reactions were conducted in triplicate. Each experiment was repeated at least three times. Glyceraldehyde-phosphate dehydrogenase (*GAPDH*) was used as the internal control as indicated and fold changes were calculated by the 2^-ΔΔCt^ method. Primers were designed and synthesized at Beijing Tsingke Biological technology (Beijing, China). Primers used in the RT-qPCR are shown in Table 1.

**Table 1:** Sequences of primers used in the qRT-PCR

Gene	Primer (5′ → 3′)	Product size (bp)	Annealing temperature (°C)
*FOXO1*	F: TTTGCCCCAGATGCCTATAC	114	57.5
	R: GGAGAGTCAGAAGTCAGCAAC		
*Bax*	F: TTTCCGAGTGGCAGCTG	74	55.8
	R: CAAAGTAGAAAAGGGCGACAAC		
*Bcl-2*	F: GGATGCCTTTGTGGAACTGT	135	57.4
	R: CACTTGTGGCTCAGATAGGC		
*caspase-9*	F: TAACAGGCAAGCAGCAAAGT	139	53.4
	R: ACCAAATCCTCCAGAACCAA		
*caspase-3*	F: AGAACTGGACTGTGGCATTG	111	55.4
	R: TAACCAGGTGCTGTGGAGTA		
*GAPDH*	F: ACGGATTTGGTCGTATTGGG	214	57.4
	R: CGCTCCTGGAAGATGGTGAT		

### Western blotting

Total protein was isolated from GCs using the RIPA lysis buffer (KeyGen Biotech. Co., Ltd.) containing Halt™ Protease Inhibitor Cocktail (Invitrogen, Karlsruhe, Germany) according to the manufacturer’s instructions. Total protein concentration was determined using a quantitative BCA protein kit (Thermo Scientific). The total proteins (60 μg/lane) were subsequently subject to 10% sodium dodecyl sulfate–polyacrylamide gel electrophoresis and transferred onto polyvinylidene fluoride membranes (EMD Millipore, Billerica, MA, USA). The membranes were then blocked with Tris-buffered saline with Tween-20® that contained 5% bovine serum albumin (Bio-Rad) for 1 h at room temperature and subsequently incubated with a primary antibody according to the manufacturer’s instructions at 4 °C overnight. Specific primary antibodies included PI3K (1:2000; ab140307, Abcam, Cambridge, MA, USA), p-PI3K (Tyr607, 1:1000; ab182651, Abcam), Akt (1:10000; ab179463, Abcam), p-Akt (Ser473, 1:2000; ab81283, Abcam), FOXO1 (1:1000; 2880, Cell Signaling, Beverly, MA, USA), p-FOXO1 (Ser 256, 1:1000; 9461, Cell Signaling), Bax (1:1000; 5023, Cell Signaling), Bcl-2 (1:500; 01556, Wanleibio, Shenyang, China), caspase-9 (1:1000; 9502, Cell Signaling), cleaved caspase-9 (Asp330, 1:1000; 7237, Cell Signaling), caspase 3 (1:1000; ab32351, Abcam), cleaved caspase-3 (Asp175, 1:1000; 9661, Cell Signaling), and GAPDH (1:2000; 2188R, Bioss, Beijing, China). On the day after washing, the membranes were incubated with secondary antibodies for 2 h at room temperature. SuperSignal® West Pico Trial Kit (ThermoFisher Scientific) was used for signal detection and the protein bands were visualized using a GelDoc XR densitometer (Bio-Rad). The relative intensities of each protein band were determined using the GAPDH band as an internal reference.

### Concentrations of active caspase-9 and caspase-3 in GCs were measured by enzyme-linked immunosorbent assay (ELISA)

The cleaved caspase-9 and caspase-3 have bioactivity to induce apoptosis. The concentrations of active caspase-9 and caspase-3 in GCs lysates were determined using human caspase-9 ELISA kit and caspase-3 ELISA kit (Elabscience Biotechnology Co., Ltd., Hubei, China), respectively, and a 450-nm Perlong DNM-9602G microplate spectrophotometer (Beijing Perlong New Technology Co., Ltd., Beijing, China) according to the manufacturer’s instructions. The amount of protein loaded in each well was the same (100 μg/well) and each sample was detected in duplicate. The intra- and inter-assay coefficients of variation for these values were < 5% and < 10%, respectively. The sensitivities of the caspase-9 and caspase-3 assays were 0.99 ng/mL and 0.19 ng/mL, respectively.

### Statistical analysis

All data were statistically analyzed using SPSS 17.0 software (SPSS Inc., Chicago IL, USA). Continuous variables are expressed as means ± standard deviation. The normality of data distribution was assessed using Kolmogorov–Smirnov tests. Between-group comparisons were assessed using one-way ANOVA with *post-hoc* Bonferroni tests. Categorical data were compared using Chi-squared tests. Two-tailed *P* values < 0.05 were considered statistically significant.

## Results

### Clinical, endocrine, and metabolic characteristics of the patients

The prevalence of irregular menstrual cycles, hirsutism, and acne in patients with PCOS was more common than in non-PCOS controls. The anthropometrics, endocrine, and metabolic parameter such as WHR, LH/FSH ratio, TT, SHBG, FAI, FINS, and HOMA-IR were significantly different in patients with PCOS when compared to non-PCOS controls (*P* < 0.05). The clinical, endocrine, and metabolic characteristics were not significantly different between the PCOS-GH and PCOS-C groups (*P* > 0.05). (Table 2).

**Table 2:** Clinical, endocrine, and metabolic characteristics of study population

	Non-PCOS (***n*** = 32)	PCOS-C (***n*** = 31)	PCOS-GH (***n*** = 30)
Age (yrs)	29.59 ± 3.02	28.90 ± 2.86	28.17 ± 3.52
Irregular menstrual cycle (*n*) ^a,b^	0	25	23
Hirsutism (*n*) ^a,b^	0	7	6
Acne (*n*) ^a,b^	0	16	16
BMI (kg/m^2^)	21.76 ± 2.50	22.12 ± 3.13	23.10 ± 2.27
WHR ^a,b^	0.82 ± 0.05	0.85 ± 0.05	0.87 ± 0.06
LH/FSH ratio ^a,b^	1.08 ± 0.56	1.55 ± 0.90	1.64 ± 1.00
E_2_(pg/ml)	46.52 ± 12.25	49.00 ± 13.33	45.14 ± 14.84
P (ng/ml)	0.52 ± 0.27	0.57 ± 0.23	0.45 ± 0.21
TT (ng/ml)^a,b^	0.49 ± 0.16	0.65 ± 0.32	0.66 ± 0.25
SHBG (nmol/l) ^a,b^	83.43 ± 25.24	42.64 ± 25.02	48.09 ± 22.11
FAI ^a,b^	2.13 ± 0.67	7.22 ± 4.23	7.13 ± 6.64
FPG (mmol/l)	4.80 ± 0.41	5.04 ± 0.52	5.11 ± 0.63
FINS (mIU/l) ^a,b^	8.11 ± 2.57	11.51 ± 6.52	11.51 ± 6.40
HOMA-IR ^a,b^	1.76 ± 0.70	2.54 ± 1.37	2.65 ± 1.55

Data are presented as mean ± SD or number (percentage). *Abbreviations*: *BMI* body mass index, *WHR* waist-to-hip ratio, *FSH* follicle stimulating hormone, *LH* luteinizing hormone, *E*_*2*_ estradiol, *P* progesterone, *TT* total testosterone, *SHBG* sex hormone binding globulin, *FAI* free androgen index, *FPG* fasting plasma glucose, *FINS* Fasting insulin, *HOMA-IR* homeostatic model assessment of insulin resistance.

^a^ *P <* 0.05, non-PCOS group versus PCOS-C group

^b^ *P* < 0.05, non-PCOS group versus PCOS-GH group

### GH inhibited ROS accumulation in GCs of patients with PCOS

**Fig. 1:**
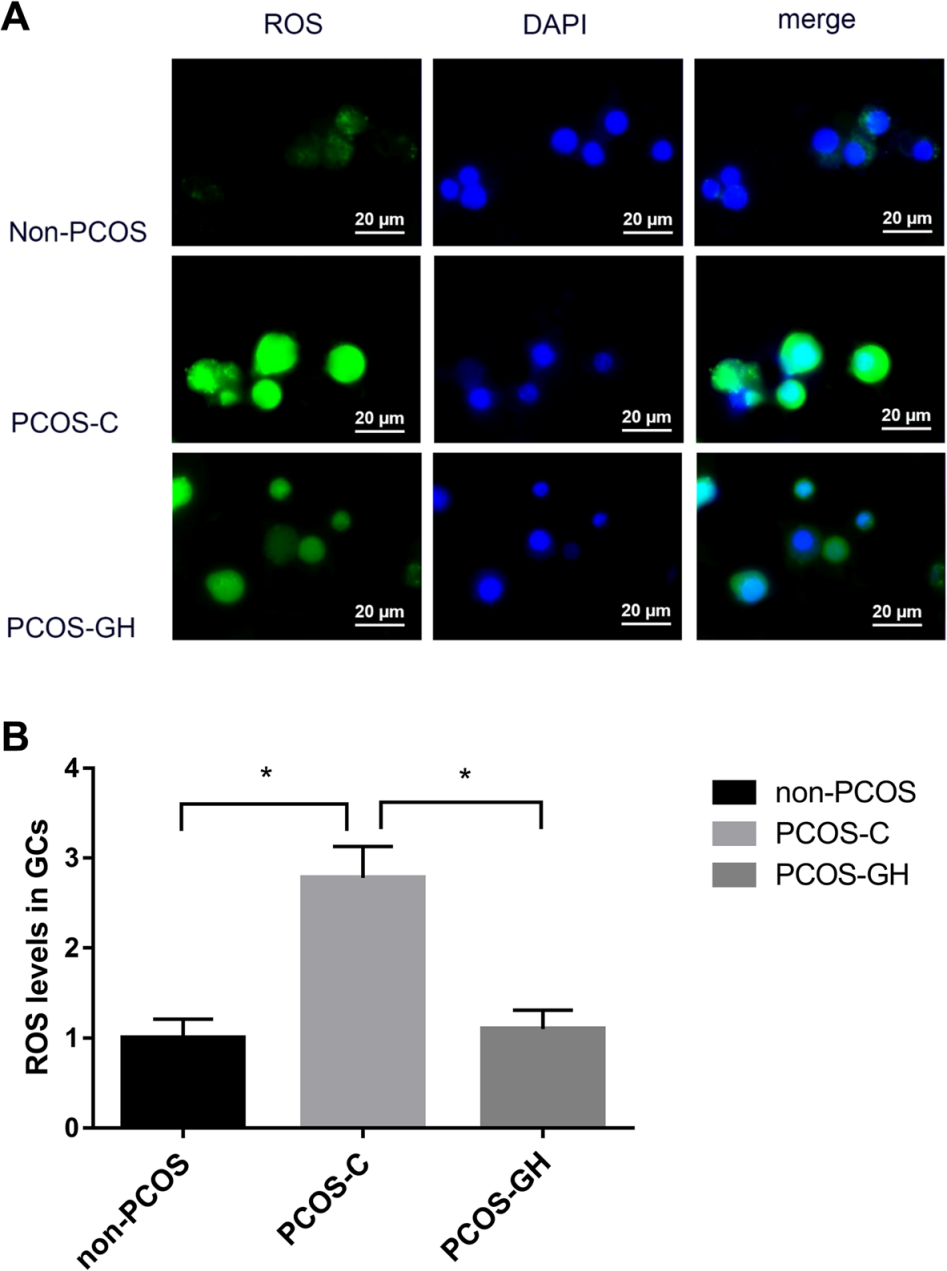
GH inhibits ROS accumulation in GCs of patients with PCOS. **a** Visualized by fluorescent microscopy, the green fluorescence intensity of ROS in GCs of the PCOS-GH group was weaker compare to that in the PCOS-C group, but similar to that of non-PCOS controls. The blue fluorescence signal indicates cell nucleus stained by DAPI. Scale bars: 20 μm. **b** Quantitative detection by using a spectrophotometer. The fluorescence intensity of ROS was expressed as the fold change relative to the control. ROS intensity was significantly lower in the PCOS-GH group than in the PCOS-C group (1.10 ± 0.21 vs. 2.78 ± 0.35) (*P* < 0.05), but no difference with non-PCOS controls (1.00 ± 0.21) (*P* > 0.05). *P < 0.05 compared with the PCOS-C group

The green fluorescence intensity visualized by fluorescent microscopy of ROS in GCs of the PCOS-GH group was weaker compare to that in the PCOS-C group, but similar to that of non-PCOS controls. Quantitative detection using a spectrophotometer indicated that the ROS intensity in the PCOS-GH group (1.10 ± 0.21) was significantly lower than that in the PCOS-C group (2.78 ± 0.35) (*P* < 0.05), but no difference with non-PCOS controls (1.00 ± 0.21) (*P* > 0.05). The fluorescence intensity of ROS was expressed as the fold change relative to the control (Fig. 1).

### GH improved MMP and inhibited GC apoptosis in patients with PCOS

**Fig. 2:**
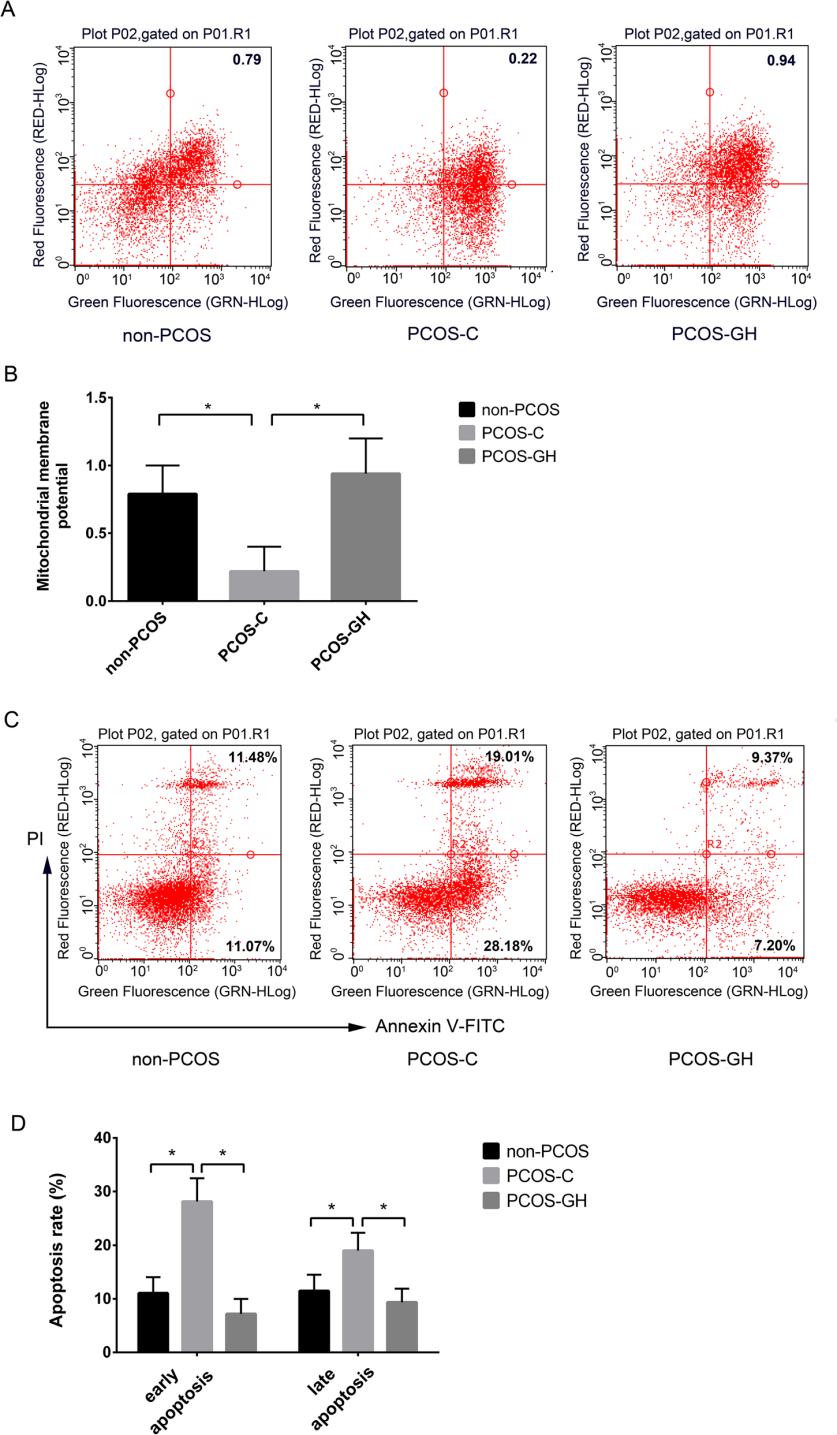
GH improves MMP and inhibits GCs apoptosis in patients with PCOS. **a** Flow cytometric dot plots showed that the ratios of red/green fluorescence are decreased in the PCOS-C group compared with those in the non-PCOS control and PCOS-GH groups. The ratios of red/green fluorescence were calculated to characterize MMP. **b** The MMP was significantly higher in the PCOS-GH group than in the PCOS-C group (0.94 ± 0.26 vs. 0.22 ± 0.18), but similar to non-PCOS controls (0.79 ± 0.21) (*P* > 0.05). *P < 0.05 compared with the PCOS-C group. **c** Flow cytometric dot plots showed that the numbers of early and late apoptotic cells are increased in the PCOS-C group compared with those in the non-PCOS control and PCOS-GH groups. PI and FITC are the abbreviations of propidium iodide and fluorescein isothiocyanate, respectively. **d** The early and late apoptotic rates were significantly lower (7.20% vs. 28.18, and 9.37% vs.19.01%, respectively) in the PCOS-GH group than those in the PCOS-C group (*P* < 0.05), but similar to those in non-PCOS controls (11.07 and 11.48%, respectively) (*P* > 0.05). *P < 0.05 compared with the PCOS-C group

MMP in the PCOS-GH group was significantly higher than that in the PCOS-C group (0.94 ± 0.26 vs. 0.22 ± 0.18) (*P* < 0.05), but no difference when compared to non-PCOS controls (0.94 ± 0.26 vs. 0.79 ± 0.21) (*P* > 0.05). (Fig. 2 a, 2 b).

The early (7.20% vs. 28.18%) and late apoptosis rate (9.37% vs.19.01%) in GCs of the PCOS-GH group was significantly lower than those in the PCOS-C group (*P* < 0.05), but similar to those in non-PCOS controls (11.07% and 11.48%, respectively) (*P* > 0.05). (Fig. 2 c, 2 d).

### GH enhanced PI3K/Akt signaling

**Fig. 3:**
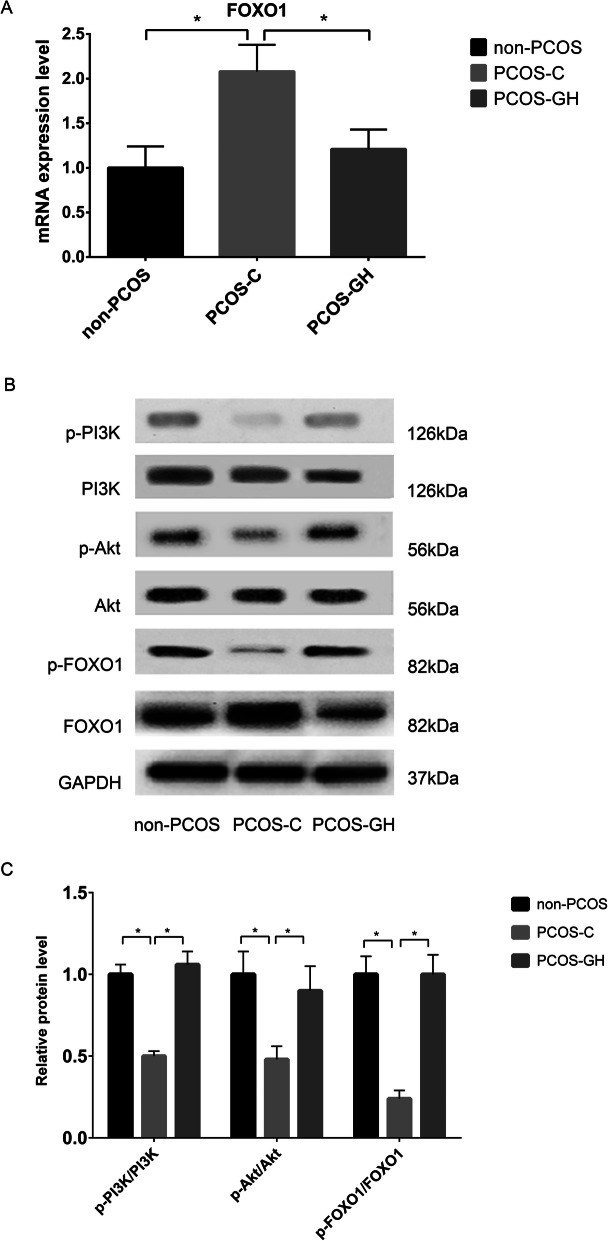
GH enhanced PI3K/Akt signaling in GCs from patients with PCOS. **a** The mRNA expression of *FOXO1* was significantly lower in GCs of the PCOS-GH group compared with the PCOS-C group (*P* < 0.05), but similar to those in non-PCOS controls (*P* > 0.05). *P < 0.05 compared with the PCOS-C group. **b** B showed the protein bands of p-PI3K, PI3K, p-Akt, Akt and p-FOXO1 and FOXO1 by western blot. GAPDH was used as a protein-loading control. **C** The protein level of p-PI3K/PI3K, p-Akt/Akt and p-FOXO1 were significantly higher, whereas FOXO1 was significantly lower in GCs of the PCOS-GH group compared with those in the PCOS-C group (*P* < 0.05), but similar to those in non-PCOS controls (*P* > 0.05). *P < 0.05 compared with the PCOS-C group

To study the mechanisms of GH for alleviating OS and mitochondrial dysfunction in GCs, candidate genes and proteins involved in PI3K/Akt signaling were determined by RT-qPCR and western blotting, respectively. In the GCs of the PCOS-GH group, the mRNA and protein levels of *FOXO1* were significantly lower than those in the PCOS-C group (*P* < 0.05); the protein levels of p-PI3K/PI3K, p-Akt/Akt, and p-FOXO1 were significantly higher than those in the PCOS-C group (*P* < 0.05). The above parameters were no difference between the PCOS-GH and non-PCOS groups (*P* > 0.05). (Fig. 3).

### GH regulated apoptosis-related genes and proteins in GCs of patients with PCOS

**Fig. 4:**
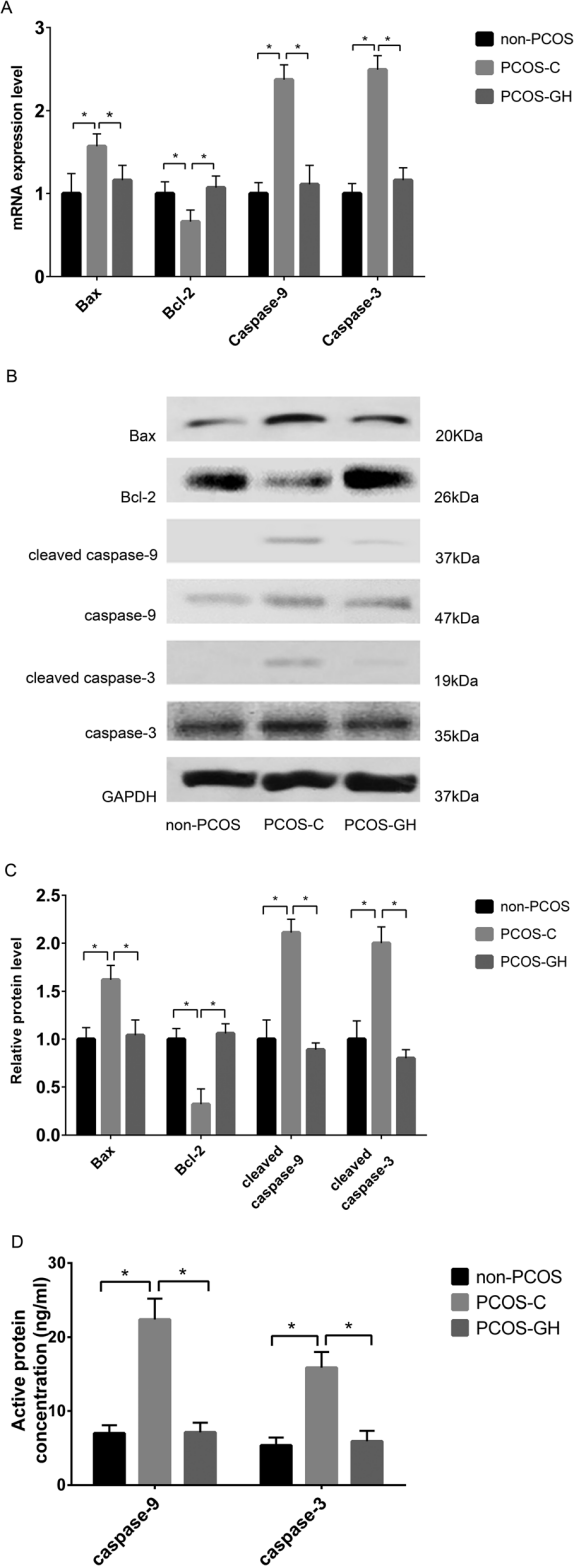
GH regulated apoptosis-related genes and proteins in GCs from patients with PCOS. **a** The mRNA expression of *Bax*, *caspase-9* and *caspase-3* were decreased, whereas that of *Bcl-2* was increased in the GCs of the PCOS-GH group compared with those in the PCOS-C group (*P* < 0.05), but similar to those in non-PCOS controls (*P* > 0.05). *P < 0.05 compared with the PCOS-C group. **b** B showed the protein bands of Bcl-2, Bax, caspase-9, cleaved caspase-9, caspase-3 and cleaved caspase-3 by western blot. GAPDH was used as a protein-loading control. **c** The protein level of Bcl-2 was increased, whereas those of Bax, cleaved caspase-9/caspase-9, and cleaved caspase-3/caspase-3 were decreased in GCs of the PCOS-GH group compared with those in the PCOS-C group (*P* < 0.05), but similar to those in non-PCOS controls (*P* > 0.05). *P < 0.05 compared with the PCOS-C group. **d** Concentrations of active caspase-9 (7.11 ± 1.31 ng/mL vs. 22.39 ± 2.79 ng/mL) and active caspase-3 (5.90 ± 1.42 ng/mL vs. 15.88 ± 2.11 ng/mL) were significantly lower in the GCs of the PCOS-GH group compared with those in the PCOS-C group (*P* < 0.05), but similar to those in non-PCOS controls (6.99 ± 1.08 ng/mL and 5.35 ± 1.06 ng/mL) (*P* > 0.05). *P < 0.05 compared with the PCOS-C group

Figure 4 showed the significantly decreased both mRNA and protein levels of *Bax*, and increased *Bcl-2* in GCs of the PCOS-GH group compared with those in the PCOS-C group (*P* < 0.05). Furthermore, both mRNA and protein levels of *Bax* and *Bcl-2* were no difference between the PCOS-GH and non-PCOS groups (*P* > 0.05).

Upon apoptotic stimulation, caspase-9 and its down signal caspase-3 is activated and cleaved, resulting in apoptosis. Figure 4 showed significantly decreased *caspase-9* and *caspase-3* mRNA levels, and decreased cleaved caspase-9/caspase-9 and cleaved caspase-3/caspase-3 protein levels in GCs of the PCOS-GH group compared with those in the PCOS-C group (*P* < 0.05). The mRNA and protein levels did not differ between the PCOS-GH and non-PCOS groups (*P* > 0.05).

Fig. 4 B showed that the protein bands of cleaved caspase-9 and cleaved caspase-3 were decreased to almost undetectable levels in the PCOS-GH and non-PCOS groups. For quantitative analysis, we measured the concentration of active caspase-9 and active caspase-3 in the cell lysate by ELISA. In Fig. 4 D, the concentrations of active caspase-9 (7.11 ± 1.31 ng/mL vs. 22.39 ± 2.79 ng/mL) and active caspase-3 (5.90 ± 1.42 ng/mL vs. 15.88 ± 2.11 ng/mL) were significantly lower in GCs of the PCOS-GH group compared with those in the PCOS-C group (*P* < 0.05). The concentration of active caspase-9 (7.11 ± 1.31 ng/mL vs. 6.99 ± 1.08 ng/mL) and active caspase-3 (5.90 ± 1.42 ng/mL vs. 5.35 ± 1.06 ng/mL) did not significantly differ between the PCOS-GH and non-PCOS groups (*P* > 0.05)

## Discussion

Ovarian antioxidants are numerous, and OS occurs when the natural antioxidant system cannot balance excessive ROS. During COS, ROS accumulates with accelerated metabolic rates for more energy and nutrients [29]. The mechanism involved in the generation of OS in PCOS still remains elusive. Our study revealed that the intracellular ROS level in GCs was increased by almost threefold and reflected that OS was overactive in patients with PCOS. Excessive ROS leads to mitochondrial dysfunction and apoptosis of GCs [8, 30]. In this study, both of early and late apoptotic rates of GCs increased about two times; MMP decreased by 72% in patients with PCOS. This reflected that excessive ROS generation may trigger opening of the mitochondrial permeability pores, thereby causing apoptosis. The result is in keeping with previous reports [8, 22]. GCs are steroidogenic cells surrounding the oocyte, which play an important role in oocyte maturation, fertilization, and subsequent implantation [8]. The apoptotic GCs may impair oocyte quality, and induce low rates of fertilization and pregnancy in patients undergoing IVF-ET [22, 31].

The PI3K/Akt pathway and the downstream pro-apoptosis genes including *FOXO1*, *Bax*, *caspase-9* and *caspase-3* play a crucial role in the regulation of GCs growth and apoptosis during follicular development [9–11]. *Bcl-2* is one of the anti-apoptosis genes. The homodimers of *Bcl-2* associate with the mitochondrial membrane and stabilize MMP. Upon apoptotic stimulation, *Bax/Bcl-2* heterodimer decrease MMP, increase membrane’s permeability and release cytochrome c, and then activate caspase family. In this study, we found that the expression of *FOXO1*, *Bax*, *caspase-9* and *caspase-3* were increased, whereas *PI3K*, *Akt*, and *Bcl-2* were decreased in GCs of patients with PCOS. The results suggested that the balance between pro-apoptosis and anti-apoptosis in GCs was lost during COS in patients with PCOS, with possible involvement of the PI3K/Akt signaling [24, 25]. PI3K/Akt signaling pathway is complicated that dependent on different cells and conditions. Our study revealed that OS-related apoptosis in GCs of patients with PCOS was accompanied with downregulated PI3K/Akt signaling and dysregulated apoptosis related genes under the condition of COS. However, the PI3K/Akt signaling was over-activated in patients with PCOS in some studies [32, 33]. The difference of the results may be attributed to the ethnic difference and research conditions.

GH plays antioxidant functions in some types of cells like oocytes, vascular endothelial cell, cardiomyocytes, neural, and skeletal muscle cells [17–20, 34]. GH was demonstrated to have both direct effects mediated by the explicit GH-GH receptor (GHR), and indirect effects through the local production of insulin-like growth factor I (IGF-I). IGF-I binds its cell-surface receptor and activates the insulin receptor substrate (IRS). The expression of GH/IGF-I and their complementary receptors have been detected in GCs [35]. This means that the GH/IGF-I system is likely to have profound effects on GCs [36, 37].

In this study, we found that GH apparently decreased ROS production by > 50%, and significantly increased MMP and lowered the early and late apoptotic rates in patients with PCOS. The mechanisms by which GH alleviates OS may involve in the PI3K/Akt pathway [17–19]. GH/IGF-I bind their cell-surface receptors and activate IRS [35]. Consequently, PI3K produces PI-3,4,5-trisphosphate (PIP3) and phosphorylates Akt, p-Akt then phosphorylates FOXO1 [35]. GH downregulates Bax and upregulates Bcl-2 by p-Akt and p-FOXO1 [38]. In skin tissue and motoneuronal, studies also reported that GH could upregulate Bcl-2 and downregulate Bax [17, 39]. Bax and Bcl-2 are apoptosis related proteins that connect with outer mitochondrial membrane. Increased levels of Bcl-2 homodimer stabilize the permeability of mitochondrial membrane [14]. Furthermore, the caspase cascade is blocked and apoptotic rate is decreased. We also found that GH apparently improved the expression of *PI3K*, *Akt* and *Bcl-2*, decreased *FOXO1*, *Bax*, *caspase-9* and *caspase-3*. Therefore, activated PI3K/Akt signaling may be one of the mechanisms by which GH may alleviate OS-associated apoptosis in GCs.

In this study, we found increased ROS levels and apoptotic rates, decreased MMP and PI3K/Akt signaling pathway, and abnormal apoptosis-associated gene and protein levels in the GCs of patients with PCOS who underwent IVF. GH administered in vivo markedly alleviated OS related apoptosis and activated PI3K/Akt signaling. To the best of our knowledge, this is the first report that GH alleviated mitochondrial dysfunction, OS-associated apoptosis and activated the PI3K/Akt signaling pathway in GCs. However, the precise mechanism that GH alleviates OS in patients with PCOS remains unclear, and further basic investigations at the cellular level in vitro and in vivo are needed.

## Conclusion

In conclusion, this study demonstrated the presence of OS state, mitochondrial dysfunction, apoptosis and downregulated PI3K/Akt signaling in the GCs of patients with PCOS undergoing IVF. GH administered in vivo markedly alleviated OS related apoptosis and activated PI3K/Akt signaling.

## Data Availability

The datasets used and/or analysed during the current study are available from the corresponding author upon reasonable request.
